# Genetic impairment of parasite myosin motors uncovers the contribution of host cell membrane dynamics to *Toxoplasma* invasion forces

**DOI:** 10.1186/s12915-016-0316-8

**Published:** 2016-11-09

**Authors:** Marion Bichet, Bastien Touquet, Virginie Gonzalez, Isabelle Florent, Markus Meissner, Isabelle Tardieux

**Affiliations:** 1 Institute for Advanced Biosciences, Team Cell & Membrane Dynamics of Host-Parasite Interactions, INSERM U1209, 38000 Grenoble, France; 2 CNRS UMR5309, 38000 Grenoble, France; 3 Université Grenoble Alpes, 38000 Grenoble, France; 4 Institut Cochin, INSERM U1016, CNRS, UMR8104, Université Paris Descartes, Sorbonne Paris Cité, 75015 Paris, France; 5 Museum National d’Histoire Naturelle, CNRS UMR7245, Sorbonne Université, 75005 Paris, France; 6 Wellcome Trust Centre for Molecular Parasitology, Institute of Infection, Immunity and Inflammation, University of Glasgow, Glasgow, UK

**Keywords:** Membrane dynamics, Cortical actin dynamics, Myosins, Forces, Cell invasion, Macropinocytosis, Protozoan parasite, Toxoplasma

## Abstract

**Background:**

The several-micrometer-sized *Toxoplasma gondii* protozoan parasite invades virtually any type of nucleated cell from a warm-blooded animal within seconds. *Toxoplasma* initiates the formation of a tight ring-like junction bridging its apical pole with the host cell membrane. The parasite then actively moves through the junction into a host cell plasma membrane invagination that delineates a nascent vacuole. Recent high resolution imaging and kinematics analysis showed that the host cell cortical actin dynamics occurs at the site of entry while gene silencing approaches allowed motor-deficient parasites to be generated, and suggested that the host cell could contribute energetically to invasion. In this study we further investigate this possibility by analyzing the behavior of parasites genetically impaired in different motor components, and discuss how the uncovered mechanisms illuminate our current understanding of the invasion process by motor-competent parasites.

**Results:**

By simultaneously tracking host cell membrane and cortex dynamics at the site of interaction with myosin A-deficient *Toxoplasma*, the junction assembly step could be decoupled from the engagement of the *Toxoplasma* invasive force. Kinematics combined with functional analysis revealed that myosin A-deficient *Toxoplasma* had a distinct host cell-dependent mode of entry when compared to wild-type or myosin B/C-deficient *Toxoplasma*. Following the junction assembly step, the host cell formed actin-driven membrane protrusions that surrounded the myosin A-deficient mutant and drove it through the junction into a typical vacuole. However, this parasite-entry mode appeared suboptimal, with about 40 % abortive events for which the host cell membrane expansions failed to cover the parasite body and instead could apply deleterious compressive forces on the apical pole of the zoite.

**Conclusions:**

This study not only clarifies the key contribution of *T. gondii* tachyzoite myosin A to the invasive force, but it also highlights a new mode of entry for intracellular microbes that shares early features of macropinocytosis. Given the harmful potential of the host cell compressive forces, we propose to consider host cell invasion by zoites as a balanced combination between host cell membrane dynamics and the *Toxoplasma* motor function. In this light, evolutionary shaping of myosin A with fast motor activity could have contributed to optimize the invasive potential of *Toxoplasma* tachyzoites and thereby their fitness.

**Electronic supplementary material:**

The online version of this article (doi:10.1186/s12915-016-0316-8) contains supplementary material, which is available to authorized users.

## Background

Non-professional phagocytes such as epithelial, endothelial, and fibroblast cells provide intracellular niches to a plethora of single-celled invasive microbes. The proliferation of these intracellular microbes including non-parasitic and parasitic prokaryotes or eukaryotes can cause diseases with devastating sociological or economic impact on livestock and humans. A common mechanism among the sequences that set intracellular niches for microbes is the remodeling of both the host cell lipid bilayer and the cortical cytoskeleton. This rearrangement provides the membrane and drives the force required to engulf the microbe within subcellular compartments. Most bacteria enter non-phagocytic cells within a few minutes by either “discreet zippering” phagocytosis or “conspicuous triggering” macropinocytosis, although this oversimplified categorization has been questioned [1]. The general concept distinguishes (1) the restrained host cell membrane protrusions firmly adhering to and extending like a zipper around the bacteria surface to form a tight-fitting phagosome from (2) the diffuse host cell membrane ruffles emerging at the vicinity of the bacteria once triggered by the first wave of bacterial effectors, and further shaping spacious macropinosomes/phagosomes [2, 3]. Once sealed, these subcellular compartments acquire and lose endocytic markers, thereby creating a microenvironment that bacteria have to control or escape [4]. In contrast, a group of flagellated protozoan parasites clustered in the Apicomplexa phylum has evolved a distinct strategy to colonize their host cells that relies upon both their own driving force and their ability to avoid the host cell endocytic pathway once internalized within vacuoles. Early studies on major members of this phylum, specifically *Toxoplasma gondii* and *Plasmodium* spp., have highlighted the lack of host cell contribution when the parasite invasive stages, also called zoites, actively invade their respective host cells in a process completed within a few seconds [5–8]. Invasion starts with the insertion in the host cell plasma membrane (PM), by the zoite, of a multi-subunit complex (identified as the apical major antigen 1 (AMA1)-rhoptry neck (RON) complex and possibly enlarged with the recently discovered claudin-like apicomplexa microneme protein (CLAMP) [9]. This macromolecular complex connects the two cells by forming a circular tight junction (TJ) [10–13] that will act as a door of entry. The zoite then tracts itself into a PM invagination that arises below the TJ [14] and then evolves as a non-fusogenic parasitophorous vacuole (PV) that will support zoite growth and multiplication [15]. Our recent kinematic analysis has allowed tracking of the RON complex during its secretion and assembly into the PM and its establishment of a traction bridge with the host PM and its associated cortical actin lattice [16, 17]. In this scheme, the invasive force is thought to be provided by the single-headed unconventional myosin A (MyoA) of the apicomplexan-specific myosin class XIV [8, 18, 19]. Accordingly, the general expectation was that *MyoA*-deficient *T. gondii* parasites would lose their ability to enter the host cells and would not be viable. Yet, using a conditional recombination system, it was possible to maintain *MyoA*-lacking (Δ*MyoA*) parasites in vitro [13, 20]. It was then proposed that another class XIV myosin, namely myosin C (MyoC), a spliced variant of the single gene encoding MyoB and MyoC isoforms, would compensate for MyoA loss of function [21]. On the other hand, mutants for additional components of the motor complex including actin —which cannot be compensated by paralogs being encoded by a single copy gene — were also engineered and have led to the proposal of alternative mechanisms for zoite motility and cell invasion based on zoite hydrodynamic forces [20] but which need to be validated. However, it has not been investigated so far whether a contribution from the host cell could potentially account for the residual invasiveness of *MyoA*-deficient *Toxoplasma*. Yet, phagocytosis-mediated uptake of live zoites into phagocytes followed within a few minutes by egress from the early phagosome into a second vacuole, namely the PV, has already been reported for motor-competent virulent [5] and avirulent [22] tachyzoites. We therefore decided to re-evaluate the role of *T. gondii* zoite motors during invasion by applying high resolution live and fixed imaging in conjunction with functional assays to compare how motor-competent and *MyoA*- or *MyoB/C*-deficient tachyzoites access a growth-compatible PV in non-phagocytic cells. Together, our data undoubtedly position the MyoA motor at the center of the *Toxoplasma* tachyzoite invasive force. Further, this study reveals that *MyoA*-deficient zoites enter host cells by a yet undescribed process that shares only initial features with macropinocytosis. This in-depth analysis of how motor-impaired *Toxoplasma* interact with mammalian cells to either succeed or fail at entering them calls for a new *Toxoplasma*-host cell invasion paradigm based on a balanced contribution between zoite motor engagement and host cell membrane/cortex dynamics.

## Results

### MyoA-deficient tachyzoites enter their non-phagocytic host cells with a significantly slower kinetics than MyoA^+^ tachyzoites

**Fig. 1:**
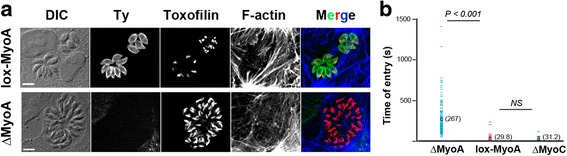
*MyoA*- but not *MyoC*-deficient tachyzoites enter non-phagocytic host cells with a significantly slower kinetics than MyoA^+^ tachyzoites. **a** *lox*-*MyoA* and Δ*MyoA* tachyzoites organized under typical rosettes inside U2OS cells after 24–40 h of intracellular growth. After fixation cells are processed for triple immunofluorescence: parasites are stained for the Ty epitope tag that is expressed in fusion with MyoA which is encoded by a loxP-flanked *MyoA* copy and for the *Toxoplasma* rhoptry protein toxofilin while the host cell F-actin cytoskeleton is visualized with fluorescent phalloidin. Maximal z projection from image stacks confirms that the Δ*MyoA* strain is specifically negative for Ty fluorescence. Scale bars: 5 μm. **b** Comparative kinetic analysis of host cell entry by *lox*-*MyoA* (*n* = 235), Δ*MyoA* (*n* = 300), and Δ*MyoC* (*n* = 200) tachyzoites. Entry duration is evaluated based on the first and last frames in which the zoite displays a clear body constriction that signs for the TJ. Statistics are performed with the Fisher test, *P* value is shown. *NS* not significant

*T. gondii* tachyzoites deficient for MyoA motor have been genetically engineered using the diCre-lox site-specific recombination system [23]. The parental line expressed a loxP-flanked sequence of *MyoA* in fusion with the Ty epitope tag (*lox-MyoA*) that ensured constitutive MyoA synthesis, while the mutant line (Δ*MyoA*) did not produce MyoA proteins as illustrated with in situ staining with anti-Ty antibodies (Fig. 1a). The duration of the entry process by tachyzoites from these two strains in a variety of non-phagocytic cells including fibroblastic, epithelial, endothelial, and osteoblastic cells was measured. While the time range was restricted for *lox-MyoA* (*n* = 235) with values rarely above 60 s (average time *x* = 29.8 s), it significantly increased for Δ*MyoA* tachyzoites (*n* = 300) with kinetic values between 64–1413 s and an average time of *x* **=** 267 s. In addition, we included another *MyoA* ^+^ strain that was deficient in *MyoB/C* (characterized in [20]) (Δ*MyoB/C*, *n* = 216) and confirmed a kinetic profile significantly similar to *lox-MyoA* tachyzoites with an average time of entry of *x* = 31.2 s (Fig. 1b), thus strongly suggesting that MyoA could play a specific key function during host cell entry.

### Real-time tracking of the host cell PM and cortical actin demonstrates that MyoA- but not MyoB/C-deficient tachyzoites are pushed into host cells through actin-powered PM protrusions that encircle the zoite body

**Fig. 2:**
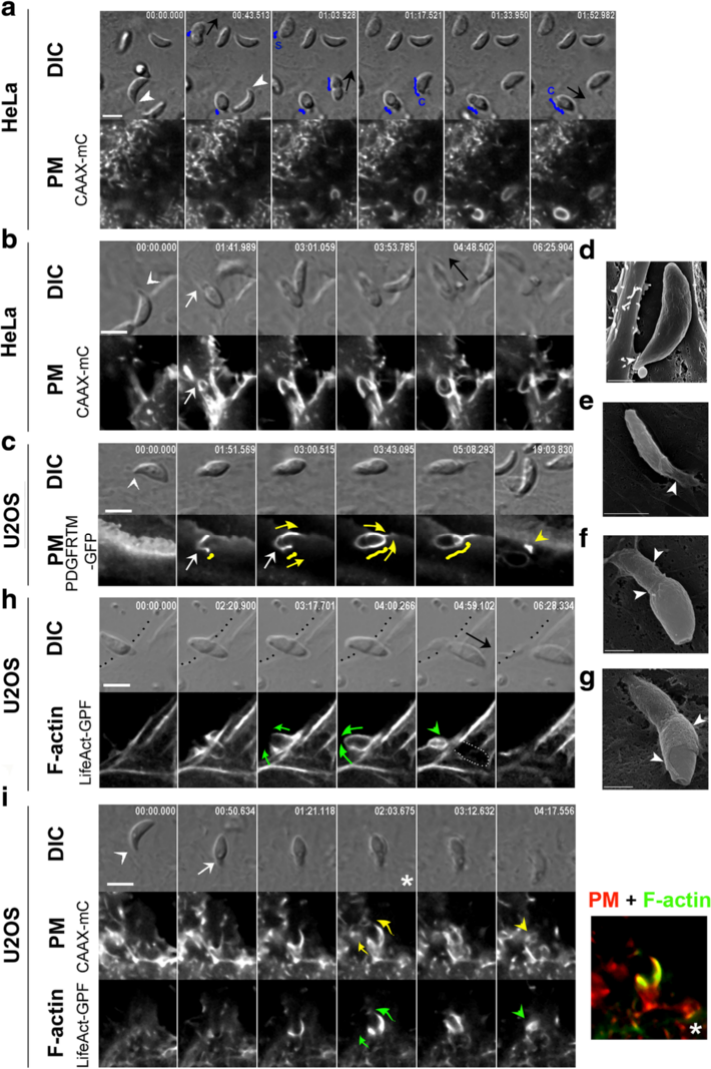
Real-time tracking of the host cell PM and cortical actin demonstrate that *MyoA*- but not *MyoC*-deficient tachyzoites are pushed into host cells through actin-powered PM protrusions. **a**–**c**, **h**, **i** Time lapses showing differential interference contrast (*DIC*) and fluorescent characteristic sequences of interaction between (**a**) Δ*MyoC* (*n* = 216) or (**b**, **c**, **h**, **i**) Δ*MyoA* (*n* = 300) tachyzoites and (**a**, **b**) HeLa cells that stably express the PM targeting domain CAAX-mCherry (*CAAX-mC*) or (**c**, **h**, **i**) U2OS cells. **c** U2OS cells transiently express the PM reporter PDGF trans-membrane domain (*PDGFTM*) coupled to GFP or (**h**) the F-actin binding peptide LifeAct coupled to GFP (*LifeAct-GFP*), or (**i**) transiently co-express the PDGFTM PM marker coupled to mCherry (*mC*) and LifeAct, coupled to GFP. Note the extruded position of the conoid prior to the contact with the host cell PM (**a**–**c**, **i**; *white arrowheads*) and the forward displacement of the tachyzoite (**a**, **b**, **h**; *black arrows*). **a** Trajectories (*blue lines*) of the constriction site (i.e., the TJ) with time reveal that Δ*MyoB/C* tachyzoites enter their host cells using a typical static or capped TJ scenario. In contrast, tracking of the PM (**b**, **c**, **i**) and cortical F-actin (**h**, **i**) dynamics reveals progressive encircling of the Δ*MyoA* tachyzoites by PM ruffles that are supported by F-actin (*yellow and green arrows*, respectively). Note that retraction of the PM ruffles together with F-actin disassembly follow zoite internalization as indicated with *yellow and green arrowheads*, respectively. **i** Right frame shows the overlap between PM and actin remodeling for the zoomed frame of the time lapse showing half in–half out zoite labeled with a *white star*. All scale bars: 5 μm. **d**–**g** SEM micrographs showing (**d**) RH-MyoA^+^, (**e**) *lox-MyoA* (MyoA^+^), (**f**, **g**) Δ*MyoA* (MyoA^-^) interacting with host cells. Note (**d**, **e**) the host cell spatially restricted “glove-like” PM protrusions around the zoite apex (*white arrowheads*) and (**f**, **g**) the thin host cell PM long sheath that constrains a third of the zoite and expands over its body. Scale bars: 1 μm (**d**, **f**, **g**) and 2 μm (**e**)


To investigate whether Δ*MyoA* tachyzoites used a distinct method of entry that would explain the slower kinetics when compared to MyoA^+^ tachyzoites, we compared the behavior of MyoA^+^ (*n* = 451) and *MyoA*-deficient (*n* = 396) parasites in contact with cells that expressed genetically encoded fluorescent markers for the PM and the nascent PV and/or for the cortical actin cytoskeleton (listed in Methods). Similarly to MyoA^+^ tachyzoites, Δ*MyoA* mutants extruded their apical conoid and made a polarized contact with the host cell PM as depicted in Fig. 2 (white arrowheads; additional movie files show this in real time (see Additional files 1–4)), but they then followed a different path. In HeLa cells expressing the CAAX-mCherry (CAAX-mC) PM reporter, *lox*-*MyoA* (= MyoA^+^) or Δ*MyoB/C* (= MyoA^+^) tachyzoites entered the nascent PVs through static or capped TJs (Fig. 2a, blue trajectories; additional movie file shows these trajectories in real time (see Additional file 5)) as described for motor-competent tachyzoites [17]. In contrast, the apex of Δ*MyoA* tachyzoites appeared tightly enwrapped within a tight collar or a glove of host cell PM protrusions we will refer to as “PM ruffles” (Fig. 2b, white arrows). In both HeLa (Fig. 2b) and U2OS (Fig. 2c) cells expressing either the CAAX-mC or the platelet-derived growth factor receptor trans-membrane domain (PDGFRTM)-green fluorescent protein (GFP) fusion proteins as PM markers, the PM ruffles were seen to protrude and stretch over the tachyzoite mid-region to eventually fold over the zoite (Fig. 2c, yellow arrows; additional movie files show this detail in real time (see Additional files 1–3)). Additional file 6: Figure S1 depicts invasion assays related to the actual actin dynamics of the PM ruffles. This is discussed in detail in a later section. PM encircling of the zoite was associated with forward displacement of the latter into the host cell (Fig. 2b, black arrows), while thereafter PM protrusions withdrew (Fig. 2c, yellow arrowheads). The sequences also revealed that once PM ruffles emerged, tachyzoites were typically pushed backward a few micrometers, suggesting that a force arose underneath the contact point. Remarkably, the PM could supply enough material to envelop two parasites that remained jointed by their posterior ends by residual material derived from the mother cell (i.e., the residual body) and which represented a volume of about 450–600 μm^3^ (an additional movie file shows this in real time (see Additional file 4)). Finally, to analyze the detailed structure of the PM protrusions that formed around the Δ*MyoA* zoites and to compare with the process induced by *lox-MyoA* tachyzoites, we used scanning electron microscopy (SEM). While we visualized thin and spatially restricted PM protrusions around the apex of MyoA^+^ attached and invading tachyzoites (Fig. 2d, e, white arrowheads), we observed a much more pronounced and PM glove-like protrusion covering the first third of the Δ*MyoA* zoite (Fig. 2f, white arrowheads) and expanding over its body (Fig. 2 g, white arrowheads) in full agreement with the dynamic imaging of mutant zoite internalization. Because cortical actin dynamics orchestrates a number of PM remodeling processes including phagocytosis and macropinocytosis [2, 24], we next investigated whether host cell actin polymerization was associated with the entry of *MyoA*-deficient zoites using cells transiently expressing the F-actin binding LifeAct peptide in fusion with GFP [25]. In U2OS cells, F-actin was detected in thick fibers throughout the cells but also in thinner structures formed at and elongated from the site of intimate contact between the two cells (Fig. 2 h, green arrows) before collapsing behind the internalized zoite (Fig. 2 h, green arrowhead; additional movie files show these details in real time (see Additional files 7 and 8)). Finally, co-expression of a fluorescent PM reporter and LifeAct-GFP in the host cell revealed the spatiotemporal coincidence of the PM (Fig. 2i, yellow arrows and arrowhead, see zoomed area) and F-actin dynamics during zoite internalization (Fig. 2i, green arrows and arrowhead; an additional movie file shows these details in real time (see Additional file 9)). All this information indicates that actin polymerization stretched and pushed the PM outwards over the zoite, thereby supporting the idea that the force directing Δ*MyoA* parasites originates from the host cell actin assembly.

Movie 1 Δ*MyoA* tachyzoite entering HeLa cell expressing the CAAX-mC PM reporter by progressive wrapping through host cell PM ruffles. Scale bar: 5 μm.
Movie 2 Δ*MyoA* tachyzoite entering U2OS cell expressing the PDFGTM-GFP PM reporter by progressive wrapping through PM ruffles. Note the retraction of the ruffles immediately behind the internalized tachyzoite. Scale bar: 5 μm.
Movie 3 Δ*MyoA* tachyzoite entering HeLa cell expressing the CAAX-mC PM reporter by progressive wrapping through PM ruffles. Scale bar: 5 μm.
Movie 4 Two attached Δ*MyoA* tachyzoite sisters engulfed in the same event by U2OS cell expressing the PDFGTM-GFP PM reporter through sustained progression of PM ruffles. Scale bar: 5 μm.
Movie 5 Δ*MyoB/C* tachyzoites invading HeLa cell expressing the CAAX-mC PM reporter through a typical static or capped TJ. Scale bar: 5 μm.
Movie 6 Δ*MyoA* tachyzoite entering U2OS cell expressing the LifeAct-GFP F-actin reporter. Note that F-actin elongates around the parasite and disassembles once internalization is complete. Scale bar: 5 μm.
Movie 7 Δ*MyoA* tachyzoite entering HeLa cell expressing the LifeAct-GFP F-actin reporter. Note that F-actin accumulates at the TJ and elongates around the parasite while disassembling once internalization is complete. Scale bar: 5 μm.
Movie 8 Δ*MyoA* tachyzoite entering HeLa cell co-expressing the CAAX-mC and LifeAct-GFP constructs. Note that PM and F-actin dynamics are tightly coupled during zoite engulfment. Scale bar: 5 μm.
Movie 9 Δ*MyoA* tachyzoite entering U2OS cell co-expressing the PDGFRTM-mC and LifeAct-GFP constructs. Note that PM and F-actin dynamics are tightly coupled in the ruffles that allow the zoite to be pushed into a PM-derived PV which is not associated with F-actin. The tachyzoite resides and further develops in this PV. Scale bar: 5 μm.


### Host cell PM ruffles force Δ*MyoA* tachyzoites to pass through the TJ into a primary PV which traffics in the cytoplasm and allows subsequent *Toxoplasma* development

**Fig. 3:**
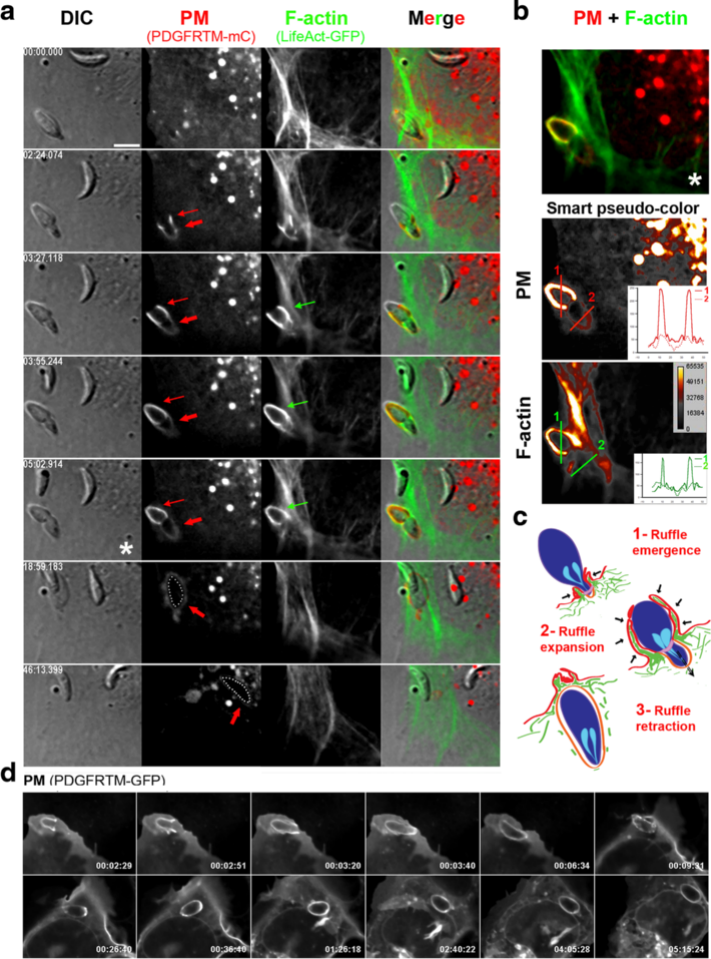
Host cell PM ruffles force Δ*MyoA* tachyzoites into a primary PV that further traffics in the cytoplasm allowing *Toxoplasma* development. **a**, **d** Time lapses showing DIC and fluorescent sequences of interaction between Δ*MyoA* tachyzoites and U2OS cells that (**b**) transiently co-express the fluorescent PM reporter PDGF trans-membrane domain (*PDGFTM*) coupled to mCherry (*mC*) and the F-actin binding peptide LifeAct, coupled to GFP (*LifeAct-GFP*) or (**d**) transiently express PDGFTM in fusion with GFP. **a** Note the concomitant development of (i) a PM invagination that remains almost F-actin-negative and that starts at the constriction site (*thick red arrows*) to eventually surround the internalized parasite and (ii) an outer basket of PM projections (*thin red arrows*) enriched in F-actin (*green arrows*). **b** Overlay between the three layers of the zoite “half in–half out” (*top frame*) and the pseudocolored image for each fluorescent signal at the same time point (*bottom frames*). Two transections covering the extracellular (1) and intracellular (2) regions of the parasites allowed us to retrieve signal intensity profiles and show that F-actin is mainly associated with the outer ruffles encircling the extracellular region of the zoite. **c** Schematic sequence of interaction events leading to progressive enwrapping of the Δ*MyoA* parasite by host cell actin-driven PM ruffles that push the zoite through the TJ. **d** Note that the zoite internalized through the wrapping process further develops in the primary vacuole over the 5-h post-entry videorecording. Scale bar: 5 μm

**Fig. 4:**
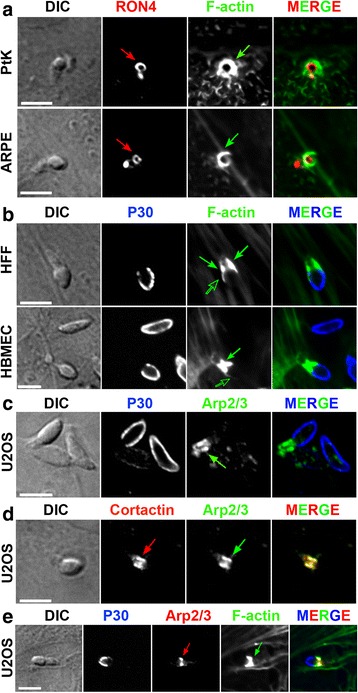
Δ*MyoA* tachyzoites form a typical ring-shaped RON-positive TJ that coincides with the strong recruitment of F-actin nucleating factors and the assembly of an actin-myosin II network. **a**–**e** Laser scan confocal micrographs of Δ*MyoA* mutant zoites in contact with (**a**) protein tyrosine kinase 1 (*PtK1*) epithelial and spontaneously arising retinal pigment epithelial (*ARPE-19*) cells, (**b**) human foreskin fibroblasts (*HFF*) and human brain microvascular endothelial cells (*HBMEC*), (**c**–**e**) U2OS osteosarcoma cells. Samples were fixed and processed for double or triple immunofluorescence staining with (**a**) anti-RON4 antibodies (*red*) and phalloidin (*green*); note the RON-positive TJ (*red arrows*), (**b**, **c**, **e**) anti-P30 antibodies (*blue*) prior to permeabilization to selectively label surface-exposed antigens, and (**b**) phalloidin (*green*) or (**c**) anti-Arp2/3 antibodies (*green*) after cell permeabilization. Note the spectacular co-enrichment of F-actin and the Arp2/3 complex that surrounded the TJ ring or area (*green arrows*), (**d**) anti-cortactin antibodies to localize the regulatory Arp2/3-nucleating factor (*cortactin, red*) and its target (the Arp2/3 complex, *green*), and (**e**) anti-Arp 2/3 antibodies (*red*) and phalloidin (*green*). Note the co-localization of F-actin and the actin nucleating machinery (Arp2/3 and cortactin) at the TJ (*green and red arrows*). All scale bars: 5 μm


Since uptake of tachyzoites through a transient phagosome has been reported [5, 22], it was pertinent to analyze in detail the fate of the Δ*MyoA* parasites once enwrapped within PM ruffles. Monitoring host cell PM dynamics allowed us to distinguish the outward collar of ruffles from a single PM invagination that hinted at a nascent PV membrane. PM invagination was detectable before ruffles expanded around the zoite and was next seen to enlarge while the zoite moved forward (Fig. 3a, red thin and thick arrows; an additional movie file shows this detail in real time (see Additional file 10)). Importantly, the signal intensity for both PM and G/F-actin markers was significantly higher for the PM ruffles than for the PM invagination (Fig. 3b, transections 1 and 2 and corresponding signal profiles). These acute observations substantiate the view of the tachyzoite being passively driven into a PM-derived PV that arose at the site of contact (see cartoon Fig. 3c). We next showed that neither escape from the primary PV nor folding of a secondary PV was required for Δ*MyoA* zoite survival and growth by monitoring vacuole-containing Δ*MyoA* mutants up to 5 h post-internalization (Fig. 3d; an additional movie file shows this detail in real time (see Additional file 11), *n* = 29 observations). Finally, to confirm that Δ*MyoA* parasites assemble a typical TJ, we applied high resolution laser scanning microscopy on samples stained for the TJ marker rhoptry neck protein (RON) 4 [10] as well as for host cell F-actin and actin-associated proteins in epithelial, fibroblastic, and endothelial cells. Not only was a typical ring-shaped RON-positive TJ (Fig. 4a, red arrows) observed in agreement with what was reported in [20], but it was surrounded by a densely packed belt of host cell F-actin (Fig. 4a, green arrows). Apart from F-actin (Fig. 4b, green arrows) the actin-nucleating factors, i.e., actin-related proteins 2 and 3 (Arp2/3) complex and cortactin (Fig. 4c–e), were largely co-recruited at the TJ site, thereby arguing for a host cell actin turnover during Δ*MyoA Toxoplasma* entry.

Movie 10 Δ*MyoA* tachyzoite entering U2OS cell expressing the PDGFRTM-GFP. Note the typical PM wrapping and the fate of the primary vacuole. The parasite remains and develops within the primary PV. Scale bar: 5 μm.
Movie 11 3D reconstruction from z stacks (2 m) capturing Δ*MyoA* tachyzoite entering an HFF cell in presence of 10 kDa fluorescent dextran (*Dx, red*). Dx serves as endosome and macropinosome markers while the ROP2 staining (*green*) indicates that the parasite has secreted the rhopty protein to enter a typical vacuole.


### The unique mode of Δ*MyoA* zoite entry relies on host cell actin polymerization but not on late effectors of macropinocytosis, consistent with the absence of macropinosome formation

Since host cell F-actin was dynamically visualized in the expanding PM protrusions enwrapping Δ*MyoA* zoites, we next interrogated how interfering with host cell actin dynamics affected the rate of Δ*MyoA* zoite entry. To this end, we exposed U2OS and spontaneously arising retinal pigment epithelial (ARPE-19) cells to a cocktail of cell-permeant actin poisons that quickly arrests actin dynamics and myosin II-based reorganization but preserves the existing steady-state actin organization, in contrast to most actin inhibitors described so far. The cocktail acts through the concerted activities of jasplakinolide, latrunculin B, and Y27632 (JLY): it is effective within seconds for periods longer than 10–15 min after drug removal [26]. These unique features gave us the opportunity to selectively block host cell actin turnover in fetal calf serum (FCS)-starved U2OS and ARPE-19 cells independently of *Toxoplasma*.

**Fig. 5:**
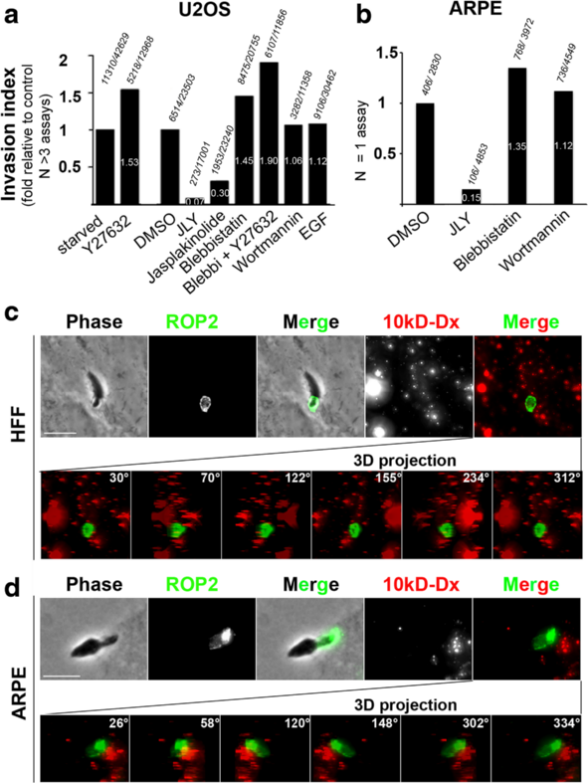
Host cell actin dynamics is required to promote Δ*MyoA* zoite entry through PM protrusions that do not promote the formation of macropinosomes but enable the folding of a typically ROP2-positive vacuole into which *Toxoplasma* is forced. **a**, **b** Graphs showing the entry rate of Δ*MyoA* zoites in (**a**) U2OS and (**b**) ARPE-19 cells. Columns represent fold of invasion by reference to appropriate control depending on the presence of DMSO or not including (**a**) 4 to 6 independent assays, and (**b**) 1 assay; all assays performed in triplicate for each parameter. Total numbers of infected cells relative to total number of scanned cells are indicated on top of each column, while the fold values are marked in *white* within each column. **c**, **d** Maximal projections of images showing tachyzoites interacting with (**c**) HFF and (**d**) ARPE-19 cells in presence of 10-kDa fluorescent dextran (*Dx, red*). Samples were stained for the parasite rhoptry resident protein (*ROP2, green*) following cell fixation and permeabilization. Frames in the bottom panels (**c**, **d**) show images taken at the indicated angles from the 3D reconstructed z stacks. Note the ROP2-positive signal in the forming vacuole surround the entering Δ*MyoA* zoite and the lack of Dx-positive signal within the PV space


We observed an acute drop in the Δ*MyoA* rate of internalization to less than 2 % (*n* infected U2OS cells = 273/17,001) in serum-starved U2OS cells treated with JLY compared to 27.7 % (*n* infected U2OS cells = 6514/23,503) when cells were incubated with the dimethyl sulfoxide (DMSO) vehicle alone (Fig. 5a; an additional file shows this assay (see Additional file 6: Figure S1a)). We did not notice any significant cytotoxic effect of the JLY by measuring the rate of live (i.e., calcein-positive) and dead (i.e., calcein-negative) cells during a period of 2 h following the JLY treatment (*n* cells = 460 to 720 cells for each measure; this assay is shown in Additional file 6: Figure S1b). In addition we observed that 2 h post-treatment, the rate of Δ*MyoA* tachyzoites was restored to more than 80 % of the control (see Additional file 6: Figure S1c). A similar decrease in internalization was obtained when ARPE-19 cells were exposed to JLY under the same experimental conditions as for U2OS cells (Fig. 5b). In the same line, when cells were pre-treated only with jasplakinolide, which blocks actin turnover by promoting the incorporation of all cytoplasmic free actin into filaments [27, 28], about a threefold decrease was observed (*n* infected U2OS cells = 1953/23,240; 8.4 %) (Fig. 5a; Additional file 6: Figure S1a shows this assay). Moreover, since myosin II also participates in the disassembly of the actin network, we used either blebbistatin, which directly inhibits myosin II activity [29], or the Rho-associated protein kinase (ROCK) inhibitor Y27632 [30], which blocks upstream myosin II activators, or a combination of the two drugs. Because each compound is soluble under different conditions and both are rapidly reversible, we used different control populations and did not wash the drug out when adding Δ*MyoA* to the pre-treated FCS-starved cells. However, intracellular mutant tachyzoites were found even at a significantly higher rate than for the respective host cell control population when myosin II activity was inhibited (for Y27632, *n* infected U2OS cells = 5218/12,968; 42 % versus control = 11310/42,629; 26. 5 % and for blebbistatin, *n* = 8475/20,755; 40.8 % versus control = 6514/23,503; 27.7 %), and an additive effect was actually obtained (Fig. 5a; Additional file 6: Figure S1a). These data indicate that host cell myosin II contractile activity was not required for the folding of membrane projections that translocated *Toxoplasma* into a nascent vacuole. Interestingly, myosin II as well as phosphoinositide 3-kinase (PI3K) have often been reported to control late steps of both constitutive (in dendritic cells) and regulated macropinocytosis or even phagocytosis [31] coinciding with ruffle closure or subsequent cup sealing [32–36]. In line with these observations, we found that pre-treatment of both U2OS and ARPE-19 cells with the irreversible PI3K inhibitor wortmannin had no effect on the rate of Δ*MyoA* parasite entry regardless of the drug concentration (*n* infected U2OS cells = 3282/11,358; 28.9 %) (Fig. 5a, b; Additional file 6: Figure S1a). In the control, uptake of the macropinocytosis tracer Alexa 594-coupled 10-kDa dextran (Dx) was nullified by all treatments (JLY, blebbistatin, and wortmannin). We also observed that the space delineated by the PM invagination around the entering Δ*MyoA* mutants was negative for the macropinocytosis tracer (*n* = 0 for >200 intracellular Δ*MyoA* tachyzoites analyzed). Collectively these data suggested that the route of Δ*MyoA* parasite entry was not macropinocytosis (Fig. 5c). We further interrogated whether a specific marker of young typical PVs such as the rhoptry protein (ROP) 2, which is thought to be secreted during invasion [37], could be associated with the process of Δ*MyoA* zoite entry. We systematically found ROP2 decorating the inner PM invagination that encircled the apical part of the Δ*MyoA* zoite, thus before complete internalization had occurred, an early and yet not documented timing for ROP2 secretion (Fig. 5c,d; an additional movie file shows this distribution (see Additional file 12)). A three-dimensional (3D) reconstruction based on image stacks suggested that the ROP2 nascent vacuole was not intimately associated with the Dx-positive compartment (i.e., endosomes and macropinosomes) (Fig. 5c, d, bottom panels; Additional file 12). In sum, these findings reinforce the live imaging data and demonstrate that Δ*MyoA* tachyzoites enter a typical PV. Note that addition of epidermal growth factor (EGF) at the time when *Toxoplasma* mutants establish contact with host cells had only a slight positive effect on parasite entry in ARPE-19 epithelial cells that elicit many ruffles in response to EGF and had no specific effect on U2OS (Fig. 5a). All these data fit well with the view that upstream of the PM remodeling, the sequences of RON release and TJ formation remain limiting steps of the entry process. Additionally these data (1) confirm that *Toxoplasma* was not internalized in a potentially harmful macropinosome but in a typical vacuole and (2) demonstrate that the force produced by host cell actin assembly drives PM protrusions.

Movie 12 Δ*MyoA* tachyzoite entering PtK1 cell. Note the extreme constriction at the TJ site. Scale bar: 5 μm.


### Long-lasting compressive forces applied by the host cell ruffles on the zoite apex induce critical hydrostatic pressure leading to *Toxoplasma* irreversible membrane damage

**Fig. 6:**
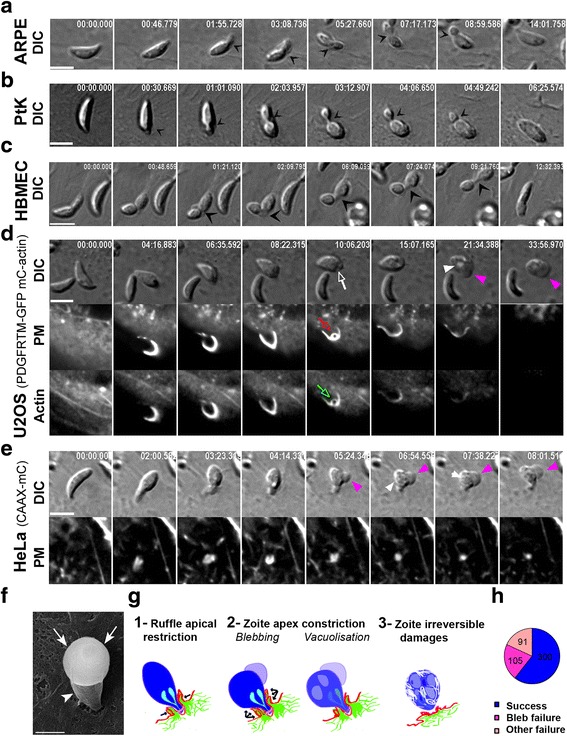
Host cell ruffles apply compressive forces on the zoite apex that critically increase zoite hydrostatic pressure leading to bleb formation with intravacuolization and eventually to zoite lysis. **a**–**e** Time lapses showing DIC and (**d**, **e**) fluorescent sequences of interaction events between Δ*MyoA* zoites and mammalian cells (**a**) ARPE-19, (**b**) PtK1, (**c**) HBMEC, (**d**) U2OS that transiently co-express the fluorescent PM reporter PDGFReceptor trans-membrane domain (*PDGFRTM*) coupled to GFP (*mC*) and the mCactin, and (**e**) HeLa cells stably expressing the fluorescent PM targeting domain CAAX-mCherry (*CAAX-mC*). **a**–**c** Black arrowheads highlight narrowness of the constriction at the TJ site that almost splits the zoite in two parts (**c**). **d**, **e** Note the equal narrowness of PM fold around the zoite apex (**d**, *red outlined arrow*) which is delineated by F-actin (**d**, *green outlined arrow*) and the release of damaged zoites. *Pink and white triangles* show the large bleb and the intravacuolization occurring while parasites are squeezed in PM. All scale bars: 5 μm. **f** SEM micrograph showing a Δ*MyoA* tachyzoite interaction with an ARPE-19 cell; note the zoite apical region strongly compressed by host cell membrane ruffles and its swollen rounded extracellular posterior end. Scale bar: 1 μm. **g** Schematic sequence of interaction events leading to irreversible zoite damage. **h** Pie graph showing the relative distribution of failed internalization events among which are those associated with a zoite membrane blebbing


A marked constriction of the zoite body was typically observed when Δ*MyoA* tachyzoites enter retinal ARPE-19 and kidney PtK1 epithelial cells (Fig. 6a, b; black arrowheads; an additional movie file shows this detail (see Additional file 13)). This constriction was exacerbated when PM ruffles persistently encaged the zoite apex without being able to progress past the TJ site, as shown in endothelial HBMEC and epithelial HeLa cells (Fig. 6c, d): in these situations, the parasite body appeared almost split into two parts from the segment of constriction (Fig. 6c, black arrowheads; an additional movie file shows this detail (see Additional file 14)). Irregular PM protrusions failed to surround tachyzoites whose apex seemed squeezed within active actin-driven protrusions (Fig. 6d, outlined arrows). Accordingly, the parasites frequently rounded up and formed a large membrane bleb (Fig. 6d, e; pink triangles) accompanied by cytoplasmic vacuolization (Fig. 6d, e; white triangles; additional movie files show these events (see Additional files 15–17)). Further, SEM analysis documented the very tight compression on the zoite anterior region trapped in the PM protrusions (Fig. 6f, arrowhead) and the swelling of the extracellular posterior part that is likely to result from the PM-driven compressive forces (Fig. 6f, white arrows) we visualized by live cell imaging. Note that this damage was concomitant with the disengagement of the zoite apex from the host cell PM, thus implying that the tight interaction between the two cells had been ruptured. The whole sequence is schematized in Fig. 6 g and accounts for about half of the internalization failures (*n* = 105/196) (Fig. 6 h).

Movie 13 Δ*MyoA* tachyzoite failing to enter HBMEC cell. Note that the zoite is almost split in two parts at the constriction site and is eventually released with marked structural alterations. Scale bar: 5 μm.
Movie 14 Δ*MyoA* tachyzoite failing to enter U2OS cell co-expressing the PDGFRTM-GFP and mC-actin constructs. Note the narrowness of the PM fold surrounding the zoite apex and supported by F-actin. As a result compressive forces are applied on the zoite and induce large membrane bleb before zoite lysis. Scale bar: 5 μm.
Movie 15 Δ*MyoA* tachyzoite failing to enter HeLa cell expressing the CAAX-mC PM reporter. Note the rearward thrust of the tachyzoite when apically engaged in a PM tunnel and the blebbing of the zoite membrane when trapped in the PM ruffles. Scale bar: 5 μm.
Movie 16 Δ*MyoA* tachyzoite failing to enter HeLa cells co-expressing the CAAX-mC and LifeAct-GFP constructs. Note that the PM tube extending beneath the zoite is enriched in F-actin. Scale bar: 5 μm.
Movie 17 Δ*MyoA* tachyzoite failing to enter HeLa cell expressing the CAAX-mC PM reporter. Note the impressive length and dynamics of the membrane tunnel that forms underneath the apex of the parasite trapped in the PM and whose membrane blebs during PM tunnel retraction. Scale bar: 5 μm.


**Fig. 7:**
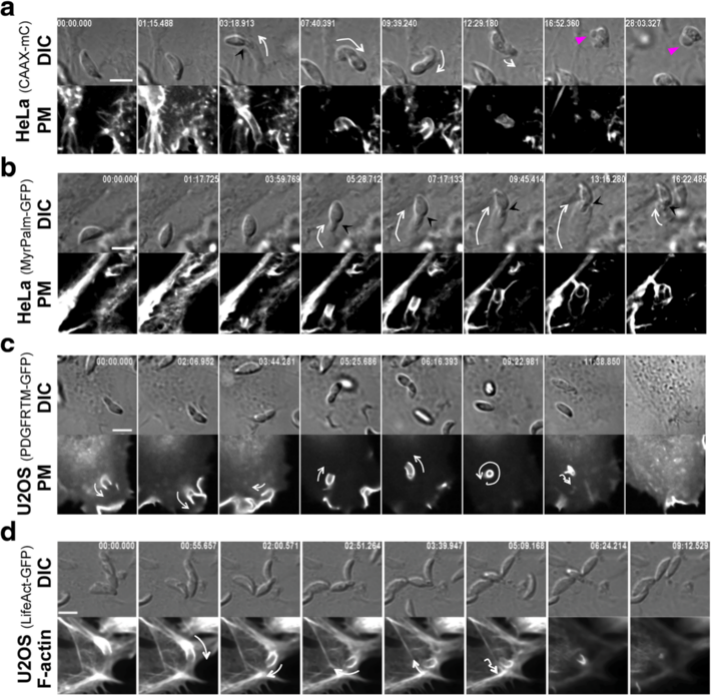
Host cell ruffles trap the zoite apex and associate with a several-micrometer-sized actin-supported stretch of the PM that rotates the zoite backward before it retracts and releases a damaged zoite. **a**–**d** Time lapses showing DIC and fluorescent sequences of interaction between *MyoA* mutants and (**a**, **b**) HeLa cells stably expressing the fluorescent PM targeting domains (**a**) CAAX-mCherry (*CAAX-mC*) or (**b**) MyrPalm-GFP, or (**c**, **d**) U2OS cells transiently expressing (**c**) the fluorescent PM reporter PDGF trans-membrane domain (*PDGFRTM*) or (**d**) the F-actin binding peptide LifeAct-GFP. Note the PM tunnel that stretches underneath the parasite and moves the parasite around: *white arrows* indicate the extension and retraction as well the rotation and bending of the PM tunnel in which the zoite apex is maintained, a process that ends with the release of damaged zoite (**a**, *pink triangles* show the large bleb formed by the trapped parasite) that is washed off under fluid flow (**c**, *last frame*). Note that F-actin is enriched at the base of the tunnel (**d**). All scale bars: 5 μm

**Fig. 8:**
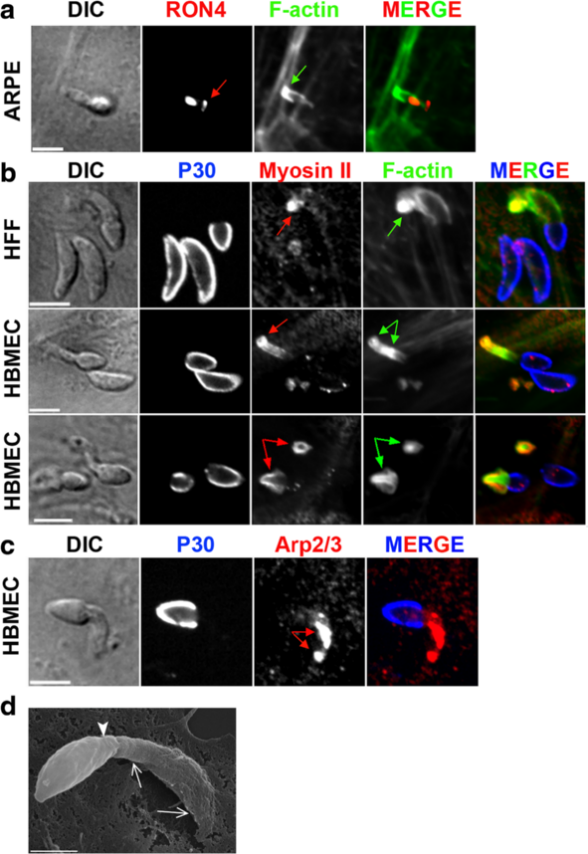
A PM tunnel folds when the Δ*MyoA* tachyzoite is attached to the cell surface through a TJ and is supported by a cap of newly assembled F-actin enriched in myosin II. **a**–**c** Laser scan confocal micrographs of Δ*MyoA* zoites in contact with HFF and HBMEC cells. Samples were fixed and processed for double or triple immunofluorescence staining with (**a**), anti-RON4 antibodies (*red*) and phalloidin (*green*): note the RON-positive TJ (*red arrow*) and the F-actin cup underneath the zoite (*green arrow*); (**b**, **c**), anti-P30 antibodies (*blue*) prior to permeabilization to selectively label surface-exposed antigens and with (**b**) anti-myosin II antibodies (*red*) and phalloidin (*green*) or (**c**) anti-Arp2/3 antibodies after cell permeabilization: note the accumulation of myosin II (**b**, *red arrows*) and F-actin (**b**, *green arrows*) at the bases of PM tubes while F-actin and the Arp2/3 complex also spread throughout the PM tube (**c**, *red arrows*). All scale bars: 5 μm. **d** Scanning electron micrograph of a Δ*MyoA* tachyzoite interacting with an ARPE-19 cell; note the impressive several micrometer-sized thin host cell PM tube-like protrusion indicated with *white arrows* into which the apical end of the parasite is embedded (*white arrowhead*). Scale bar: 1 μm


Interestingly, an impressive rearward thrust of the tachyzoite when apically engaged in the PM invagination could also be observed when PM ruffles failed to enwrap the zoite body. These vigorous movements were imposed by a several-micrometer-sized host cell PM dynamic stretch or tube supported by F-actin that we visualized using three different PM reporters (Fig. 7a–c, white arrows; additional movie files show this detail in real time (see Additional files 18–20)) and the LifeAct actin reporter (Fig. 7d; an additional movie file shows this detail (see Additional file 21)), respectively, until it retracted, further releasing tachyzoites with structural alterations (Fig. 7a, pink triangles). Even when the deformation was discrete, a short pulse of medium fluid flow was sufficient to wash the tachyzoite off the cell, thereby confirming that the attachment of the zoite to the PM had been disrupted (Fig. 7c; additional movie file shows this detail (see Additional file 20)). Next, static fluorescent imaging with anti-RON4 antibodies and phalloidin confirmed (1) that a TJ had initially formed (Fig. 8a) and (2) that host cell actin-myosin contractility was likely to contribute to PM tube formation and dynamics. Indeed a prominent accumulation of F-actin (Fig. 8a, b) together with the Arp2/3 complex (Fig. 8c) were seen to culminate at the tube base together with the myosin II motor (Fig. 8b), suggesting that a second process of PM dynamics might be coupled to the emergence of compressive PM ruffles. Remarkably, SEM images capturing the host cell responses to Δ*MyoA* tachyzoites showed an impressively large PM tube ahead of the zoite, in particular at its base (Fig. 8d, white open arrows), the apex of the latter still being maintained by the PM folds (Fig. 8d, white arrowhead).

Movie 18 Δ*MyoA* tachyzoite failing to enter HeLa cell expressing the MyrPalm-GFP PM reporter. Note the impressive length and dynamics of the membrane tunnel that forms underneath the apex of the parasite, which is trapped in the PM inner invagination and is released after tunnel retraction. Scale bar: 5 μm.
Movie 19 Δ*MyoA* tachyzoite failing to enter U2OS cell expressing the PDGFRTM-GFP PM reporter. Note the impressive length and dynamics of the PM tunnel that forms underneath the apex of the parasite, which is eventually released after tunnel retraction and is no longer attached to the cell. Scale bar: 5 μm.
Movie 20 Δ*MyoA* tachyzoite failing to enter U2OS cell expressing the LifeAct-GFP F-actin reporter. Note that the PM tunnel that folds underneath the parasite is enriched in F-actin. Scale bar: 5 μm.


## Discussion

Until recently, the force driving the entry of *Toxoplasma* tachyzoites into non-professional phagocytes was thought to exclusively rely on the functional interplay between parasite F-actin and myosin motors while the host cell remained essentially passive [5, 6]. This study alters this view by reporting that tachyzoites devoid of MyoA motor can still access a typical PV following their polarized contact with the target cell due to an active contribution of the latter. This work also highlights the need to assess in depth the contribution of host cell membrane dynamics to the high-speed process of wild-type *Toxoplasma* entry into cells.

### Host cell actin-based forces promote the formation of PM protrusions that drive a fraction of the Δ*MyoA* tachyzoite population into a typical PV through a process with unique features

In the absence of the MyoA motor, we found that tachyzoites can rely on local host cell PM protrusions that fold over the distal part of the zoite body and apply forces eventually moving the tachyzoite through a TJ into the nascent PV. Such observations explain the in vitro viability of the Δ*MyoA* mutant. Because of the drastic reduction in the rate of internalized Δ*MyoA* parasites when host cells are chemically impaired in actin polymerizing/depolymerizing machineries, we conclude that host cell actin turnover is necessary to internalize these mutants. In line with this requirement, dynamic tracking of the PM/PV and of actin cytoskeleton remodeling revealed a selective accumulation of F-actin underneath the TJ and within the PM projections wrapping around the zoite. While *Toxoplasma* survival is expected to be prevented if the PM projections sealed into a macropinosome-like compartment that would rapidly integrate the endocytic pathway [36], Δ*MyoA* parasites were indeed not found within macropinosomes. Furthermore, the lack of effect of inhibitors directed against the PI3K or against myosin II and ROCK, all known to target late effectors required for macropinosome or phagosome closure [32, 33, 38–41], allowed us to propose a novel strategy used by *Toxoplasma* tachyzoites to enter cells that shares initial but not late features with macropinocytosis.

### TJ assembly in the host cell PM/cortex associates with PM/cortex dynamic remodeling

The *MyoA-*deficient *Toxoplasma* is the first mutant providing the unique opportunity to uncouple TJ insertion into the host cell PM from zoite motor activity. In the absence of MyoA, we found that the early steps of invasion, before mobilization of motor activity, i.e., rhoptry secretion and/or TJ folding, were associated with confined host cell PM ruffle-like protrusions. The flattening out of PM reservoirs is now universally recognized as an actin assembly-driven mechanism regulated by membrane tension to adjust cell shape and properties [42, 43]. Insertion of hydrophobic proteins in the PM can act as wedges known to create local changes in curvature in tight interplay with actin rearrangements [44]. Interestingly, further characterization of several host actin regulatory factors that have been identified as required during cell invasion using high throughput RNA interference screening should enable one to shed light on the host cell cortex remodeling during *Toxoplasma* entry [45]. Therefore, we propose that the PM protrusions emerging around Δ*MyoA* mutants result from the local change of PM tension occurring upon insertion of the RON complex. In addition the local dismantling of the host cell cortical actin meshwork following secretion of the actin-severing rhoptry protein toxofilin at the onset of entry [46] is likely to contribute to a shift in membrane tension. Future engineering of a double *MyoA*-*toxofilin* knock out *Toxoplasma* would help to precisely decipher whether toxofilin activity can not only (1) fuel the TJ anchoring response as we proposed in, but also (2) be involved in more complex changes in PM dynamics including those promoting ruffle-like protrusions. If we hypothesize that TJ induces PM remodeling, we could expect the PM responses to be limited when the TJ remains transient as occurs with motor-functional tachyzoites, whereas these changes should be exacerbated in time and nature when tachyzoites have no MyoA motor to engage generation of traction forces at the TJ. Note that several SEM micrographs have documented protrusions around the apex of motor-competent zoites in endothelial and leukocyte cells [47, 48], neutrophils included [49] during what was thought to be active invasion. Importantly, while neutrophils are prototypic cells for PM unwrinkling and tube-like protrusions [50, 51], our SEM analysis revealed that fibroblasts also formed small PM ruffles around the apex of motor-competent parasites, thus reinforcing the concept of a general early and transient local response from the host cell PM and cortex when the TJ builds up regardless of subsequent motor engagement. SEM also strongly supported a key contribution of PM remodeling during internalization of *MyoA*-deficient zoites. It is worth mentioning the recent biophysical model of plasmodial merozoite invasion of the erythrocyte that assumes a modification of PM curvature triggered by the TJ insertion in the red blood cell and proposes this shift to account for the early enwrapping of the zoite [52]. The triggers underlying changes in PM might be even more diverse, as a pioneer study using optical tweezers showed that randomly attached merozoites, i.e., those that have not released RONs, were already able to induce red blood cell deformation [53]. In this framework, analyzing if and how *MyoA-*deficient *Plasmodium* merozoites triggered sufficient changes in the red blood cell PM and underlying cytoskeletal cortex to be successfully enwrapped will certainly be informative.

### Multiple and intertwined PM responses following TJ assembly can lead to either successful or failed internalization events

The membrane dynamics that operates at the TJ site includes not only the PM outer protrusions but also a tight-fitting inner PM invagination that surrounded the apex of the mutant zoite ahead from the TJ. The dynamics of the two processes worked in tandem as they concomitantly formed, retracted, and withdrew. Quantitative analysis of the video sequences revealed that when PM ruffles could not stretch enough over the zoite body, they instead trapped the zoite into the PM infolding to eventually cause a huge membrane bleb in the zoite. These observations suggest that PM outer protrusions apply compressive forces on the apex of the zoite that can harm the zoite. In support of this view is the increased rate of internalization (i.e., decrease in the rate of failed events) when host cell contractile activities are inhibited. In addition, in some abortive events, a tube of PM several micrometers long that spun and dragged the parasite formed underneath the TJ being enriched in F-actin and associated actin proteins, in particular myosin II. In that setting the zoite stayed engaged in the PM inner tight invagination and showed a RON2-positive ring that suggested a TJ had formed. Collectively these data point to a complex interplay between effector and regulatory factors of the host cell actomyosin-driven contractile activities to promote folding of the PM tube.

## Conclusions

This study positions *T. gondii* tachyzoite MyoA as a central provider of parasite invasive force and highlights a previously unseen mode of entry for intracellular microbes, relying on host cell membrane dynamics following zoite tight polarized contact. It provides a precise explanation for the marked reduction in invasiveness for Δ*MyoA* tachyzoites. Future structural and functional studies are necessary to understand how the RON complex insertion dictates the fast remodeling of the PM and how its tension/stretching properties are changed and appropriate curvatures promoted to allow (1) PV folding and (2) ruffles/tube assembly and dynamics. An interesting candidate is the CLAMP, appropriately localized to the cell zoite TJ throughout invasion, as published in the course of the reviewing process by the team of Lourido [9]. Finally, given the harmfulness of the host cell PM remodeling on the *MyoA*-deficient zoite, we propose to consider host cell invasion as the competitive contribution of both a *Toxoplasma* motor function and a complex host cell membrane dynamics. The shaping of MyoA during evolution, with a low affinity for ADP [54] that correlates with fast motor activity, may have contributed to optimize the invasive potential of *Toxoplasma* tachyzoites and thereby their fitness.

## Methods

### Cells, parasites, and reagents

All media and products used for cell culture were from Gibco-Life Technologies (St Aubin, France) unless specified. Cells in culture were strictly screened for mycoplasma contamination monthly with the MycoAlert Kit (Lonza Rockland, Rockland, ME, USA) and cured with Plasmocin™ (InvivoGen, San Diego, CA, USA) if necessary. Human foreskin fibroblasts (HFFs), human epithelial cervical cancer cells (HeLa), human ARPE-19 retinal epithelial cells, and human U2OS osteosarcoma epithelial cells were grown in Dulbecco’s modified Eagle’s medium (DMEM) supplemented with GlutaMAX, 10 % heat-inactivated FCS, penicillin (100 U/mL), streptomycin (100 mg/mL), and 10 mM 4-(2-hydroxyethyl)-1-piperazineethanesulfonic acid (HEPES). When specific fluorescent HeLa cell lines were used, they were grown in the presence of the appropriate antibiotics (puromycin or G418) used for selection: these lines stably express either the lipid- (non raft) CAAX or the lipid- (raft) MyrPalm PM targeting domain in fusion with mCherry and GFP, respectively [17]. Rat kangaroo kidney epithelial cells (PtK1) were cultured in Ham’s F12 medium (Sigma-Aldrich, Lyon, France) containing 25 mM HEPES, 10 % fetal bovine serum (FBS), and antibiotics. Human brain endothelial cells (HCMEC/D3) were grown in Endothelial Basal Medium-2 (Lonza Walkersville, Walkersville, MD, USA) supplemented with 5 % FBS, 10 mM HEPES, antibiotics, 1 % chemically defined lipid concentrate (Invitrogen Ltd., Paisley, UK), 1.4 μM hydrocortisone, 5 μg/mL ascorbic acid, and 1 ng/mL basic fibroblast growth factor (Sigma-Aldrich, St Louis, MO, USA). *T. gondii* strains (*lox*-*MyoA*, Δ*MyoA*, Δ*MyoB/C*) were propagated on HFF cells. All cultures were maintained at 37 °C and 5 % CO_2_ atmosphere. Antibodies used in this study included the homemade affinity purified rabbit anti-*T. gondii* toxofilin [55], affinity purified mouse anti-*T. gondii* RON4 antibodies [17], mouse monoclonal anti*-T. gondii* P30 antibodies (Novocastra, Leica Biosystem, Nanterre, France), polyclonal rabbit anti-p34-Arc/ARPC2 (ref 07-227 batch 32474, Upstate, Millipore, Molsheim, France), mouse monoclonal anti-cortactin p80/p85 (clone 4 F11, ref 05-180, batch 28747, Upstate, Millipore, Molsheim, France), anti-non-muscle myosin II (ref M8064, Sigma-Aldrich, Lyon, France), mouse anti-Ty antibodies (ref 200-301-W45, Rockland Immunochemicals Inc., Limerick, PA, USA), anti-recombinant ROP2 serum (gift of J.F. Dubremetz). Secondary antibodies used were highly cross-adsorbed goat anti-mouse, goat anti-rabbit, or goat anti-rat antibodies conjugated with Alexa Fluor*®* 488, Alexa Fluor*®* 568*,* Alexa Fluor*®* 633, or Alexa Fluor*®* 660 (Life Technologies, Thermo Fisher, Waltham, MA, USA). The micropinocytosis marker Alexa Fluor*®* 594 dextran (10 kDa, ref D22913) was obtained from Life Technologies, Thermo Fisher, Waltham, MA, USA. Inhibitors used in this study included the actin drugs jasplakinolide (ref J4580) and latrunculin B (ref L5288), the ROCK inhibitor Y27632 (ref Y0503), the myosin II ATPase inhibitor blebbistatin (ref B0560), the PI3K inhibitor wortmannin (ref W1628), and the DNA stain Hoechst 33258. All were purchased from Sigma-Aldrich (Lyon, France).

### Transient expression of PM and actin fluorescent reporters

In addition to the PM reporters that were stably expressed in HeLa cells (see above), we used transient expression of additional host cell PM and actin markers. U2OS and HeLa cells were routinely transfected separately or in pair combination with various constructs. The list of constructs included pDisplay^*TM*^ plasmid encoding the PDGFR trans-membrane domain (Life Technologies, Thermo Fisher, Waltham, MA, USA) in fusion with GFP (gift from V. Heussler, Institut Cell Biology, Bern (CH), Bern, Switzerland) or in fusion with mCherry (mC) (homemade), p^CMV^mCherry-actin (gift of V. Delorme-Walker, Scripps, La Jolla, CA, USA), and p^CMV^LifeAct-TagGFP2 (Ibidi, Biovalley, Nanterre, France).

### Videomicroscopy, confocal microscopy, and image acquisition

Parasites were collected within a few hours following spontaneous egress from the HFF monolayers and washed in Hanks’ Balanced Salt Solution (HBSS) supplemented with 1 % FCS (HBSS-FCS). Time-lapse video microscopy was conducted in Chamlide chambers (LCI Corp., Seoul, Korea) installed on an Eclipse Ti inverted confocal microscope (Nikon France Instruments, Champigny sur Marne, France) with a temperature- and CO_2_-controlled stage and chamber (LCI Corp., Seoul, Korea), equipped with a CoolSNAP HQ2 camera (Photometrics, Roper Scientific, Lisses, France) and a CSU X1 Yokogawa spinning disk (Roper Scientific, Lisses, France), three lasers (with excitation wavelength λ 491, 561, and 642 nm), and three dichroic mirrors. The microscope was piloted using Metamorph software (Universal Imaging Corporation, Roper Scientific, Lisses, France), and images of parasite-cell interaction were acquired with settings including 1 or 2 frames/s for up to 40 min, and 1 frame/30 s for up to 6 h when assessing the viability of internalized parasites. Depending on the experiment, one to two laser wavelengths were used sequentially for each time point to monitor separately or simultaneously the dynamics of PM and actin fluorescent reporters. When needed, the chamber was perfused with syringe-pumped HBSS-FCS medium for 1 min at a medium flow rate of about 20–30 μL/min. Confocal imaging was performed on the same device using the three lasers with sections ranging from 0.250–0.3 μm.

### Image analysis

Image stacks for every event of interest were prepared and annotated with time, bar scale, and arrows with Metamorph software from the raw image data file. Next ImageJ software (Rasband, W.S., ImageJ, US National Institutes of Health, Bethesda, MD, USA, http://imagej.nih.gov/ij/, 1997–2014) was used to add some additional labels on the time lapses, movies, and maximal projections from confocal z stacks. In some video sequences, the “Manual tracking” plug-in was used to track in time the spatial *xy* positions of the parasite’s constriction site [17].

### Immunofluorescence labeling of Δ*MyoA* tachyzoites interacting with host cells

We analyzed the modalities of interaction between Δ*MyoA* parasites and a variety of epithelial, fibroblastic, endothelial, and osteosarcoma cells that were grown in complete medium at 70–90 % confluency on poly-L-lysine-coated glass coverslips. Cell monolayers were washed with HBSS-FCS, and newly released Δ*MyoA* parasites were rapidly centrifuged (1.5 min, 250 g) on top of the monolayers to synchronize the contact between the two partner cells. Samples were next incubated for 10–20 min under culture conditions before paraformaldehyde (PFA) fixation (2 % in phosphate-buffered saline (PBS), 30 min, room temperature (RT)). Free aldehydes were quenched in NH_4_Cl (50 mM, 10 min), and cells were incubated in blocking buffer (2 % bovine serum albumin in PBS, 30 min, RT), then with anti-P30 antibodies (20 min, RT) (Novocastra, Nanterre, France) followed by Alexa Fluor*®* conjugated anti-mouse antibodies (30 min, RT) (Molecular Probes, Life Technologies, St Aubin, France). Samples were next permeabilized with 0.5 % Triton X-100 (5 min, RT), incubated again with the blocking buffer and then with Alexa Fluor*®* conjugated phalloidin to stain F-actin (2 μM, 45 min, RT) or sequentially with antibodies against proteins of interest (see list above) followed by relevant Alexa Fluor*®* conjugated secondary antibodies (1 h, RT). In some assays, cell permeabilization was performed straight after fixation prior to immunostaining. In the assays performed with both Δ*MyoA* tachyzoites and the fluorescent Alexa^594^ dextran (500 μg/mL, 10 kDa), ARPE-19 and HFF cells were fixed after 10 or 20 min of interaction and permeabilized with Triton X-100 (0.01 % in PBS, 5 min, RT). Mouse serum against ROP2 and anti-mouse Alexa Fluor*®* conjugated secondary antibodies were used to stain the PV membrane [37]. Cells were mounted in Mowiol® 4-88 (Sigma-Aldrich, St Louis, MO, USA) and analyzed within 24 h by confocal microscopy using the Eclipse Ti inverted microscope.

### Scanning electron microscopy

ARPE-19 cells were grown at 80 % confluency for 24 h on poly-l-lysine-coated glass coverslips and incubated with either RH-*MyoA*, *LoxMyoA*, or Δ*MyoA* tachyzoites for 4–5 min (MyoA^+^) and 15–20 min (Δ*MyoA*). After cell fixation in 2.5 % glutaraldehyde in 0.1 M cacodylate buffer (pH 7.2) (1 h, 4 °C), samples were washed in 0.1 M cacodylate buffer (pH 7.2) (12 h, 4 °C), ethanol dehydrated, and critical point dried in CO_2_ atmosphere with an Emitech K850 apparatus (Quorum Technologies, Laughton, UK). Coverslips containing the infected monolayers were attached to SEM aluminum holders and were gold coated using a JEOL SEM instrument, JPC-1200 (JEOL, Freising, Germany) and analyzed with the Scanning Electron Microscope SU3500 (Hitachi, Tokyo, Japan). Digital images were recorded, and photocompositions were realized with ImageJ and Photoshop software.

### Invasion assays

About 2 × 10^4^ U2OS and ARPE-19 cells were plated in a 96-well plate to obtain 80 % confluency 24 h later. Cells were starved in 0.01 % FCS for 12–16 h prior to the assay and were pre-treated as follows: (1) with JLY as described in [26] with 20 μM Y27632 for 10 min prior to addition of 8 μM jasplakinolide and 5 μM latrunculin B for an additional 10 min before extensive washing in medium and immediate contact with Δ*MyoA* tachyzoites, (2) with jasplakinolide alone (1 μM) for 15 min and treated as indicated for JLY inhibitors, (3) with blebbistatin (25 μM), Y27632 (20 μM) separately or in combination for 15 min with the drugs kept during the invasion assays due to the high reversibility of both compounds, and (4) with wortmannin (10 μM) for 30 min that was washed out prior to the invasion assay. Δ*MyoA* tachyzoites were settled on top of the cells by gentle centrifugation (2 min, 250 g) and incubated at 37 °C and 5 % CO_2_ for 15 min before PFA fixation. Following sequential labeling of extra- and intracellular tachyzoites with anti-TgP30 and anti-TgGRA1 antibodies and differential fluorophore-coupled secondary antibodies, respectively, prior and post TX-100 permeabilization, Hoechst 33242 was added to label all nuclei (mammalian cells and parasites), and the samples were automatically scanned at a magnification of × 20 under an Olympus Scan^R automated inverted microscope (3 wells per condition, 16 fields of acquisition per well). Image processing with the Cell^R software successively included signal-to-noise ratio optimization to allow cell nuclei segmentation, channel-associated image detection, and image subtraction (extracellular zoites subtracted from extra- plus intracellular tachyzoites), and intracellular tachyzoite segmentation using an edge detection algorithm. The whole assay including imaging procedure was applied to samples of untreated pre-starved ARPE-19 and HFF cells that were incubated in complete medium supplemented with 20 % FCS but no phenol red with Δ*MyoA* tachyzoites to which Alexa 594 dextran (500 ug/mL) was added for 10 min after the parasite centrifugation step. Samples were fixed in PFA, permeabilized, and stained with anti-*T. gondii* ROP2 antibodies to observe macropinosomes and nascent PVs. Control of the efficiency of macropinocytosis inhibitors was performed under the conditions used for invasion assays and assessed by quantifying the number of macropinosomes positive for fluorescent Alexa^594^ dextran (500 μg/mL, 10 kDa) as described [56]. Statistics were performed with GraphPad Prism software. To check whether JLY treatment was cytotoxic for the U2OS cells, we assessed viability by pre-loading them with cell-permeant calcein-AM (0.5 μM final dilution) and Hoechst 33258 (1.5 μM final dilution) reagents (15 min, RT) before JLY exposure. Drugs were removed and the amount of green (i.e., live) and blue (i.e., total) fluorescent cells was measured over a 2-h period using semi-automatic imaging (×20) with an Olympus Scan^R automated inverted microscope under conditions similar to those used for the invasion assays in P96-well plates (*N* = 1 experiment). Reversibility of the JLY effect on cell invasion was measured under the same conditions (*N* = 3 experiments).

## Additional files

Additional file 6: Figure S1. (a) Histogram showing one representative invasion assay of U2OS cells by Δ*MyoA* tachyzoites under different drug treatment. Student *t* test has been applied using GraphPad Prism software. (b) Graph showing the amount of calcein-positive U2OS cells following JLY treatment and throughout a 2-h period to assess the cytotoxicity of the JLY treatment. Total cells are quantified by use of the cell permeant DNA stain Hoechst 33258. (c) Histogram showing the internalization index after normalization to control solvent conditions following (1) JLY treatment and (2) a 2-h recovery time post- JLY treatment to assess the reversibility of the drug effects. Numbers of cells infected versus total cells are indicated on top of each column. (TIF 282 kb)

## Abbreviations

AMA1: Apical major antigen 1
CLAMP: Claudin-like apicomplexa microneme protein
DMEM: Dulbecco’s modified Eagle’s medium
DMSO: Dimethyl sulfoxide
Dx: Dextran
EGF: Epidermal growth factor
FCS: Fetal calf serum
HCMEC: Human brain endothelial cells
HEPES: 4-(2-hydroxyethyl)-1-piperazineethanesulfonic acid
HFF: Human foreskin fibroblast
JLY cocktail: Jasplakinolide-latrunculin-Y27632 cocktail
mC: mCherry
MyoA: Myosin A
MyoB: Myosin B
MyoC: Myosin C
PBS: Phosphate-buffered saline
PDGFRTM-GFP: Platelet-derived growth factor receptor trans-membrane domain-green fluorescent protein
PI3K: Phosphoinositide 3-kinase
PM: Plasma membrane
PV: Parasitophorous vacuole
ROCK inhibitor: Rho-associated protein kinase inhibitor
RON protein: Rhoptry neck protein
ROP protein: Rhoptry protein
RT: Room temperature
SEM: Scanning electron microscopy
TJ: Tight junction
