# Supplementary figures and images for: Genetic impairment of parasite myosin motors uncovers the contribution of host cell membrane dynamics to Toxoplasma invasion forces
Source: BMC Biol. 2016 Nov 9;14:97. doi: 10.1186/s12915-016-0316-8 (PMC5101828; doi:10.1186/s12915-016-0316-8)

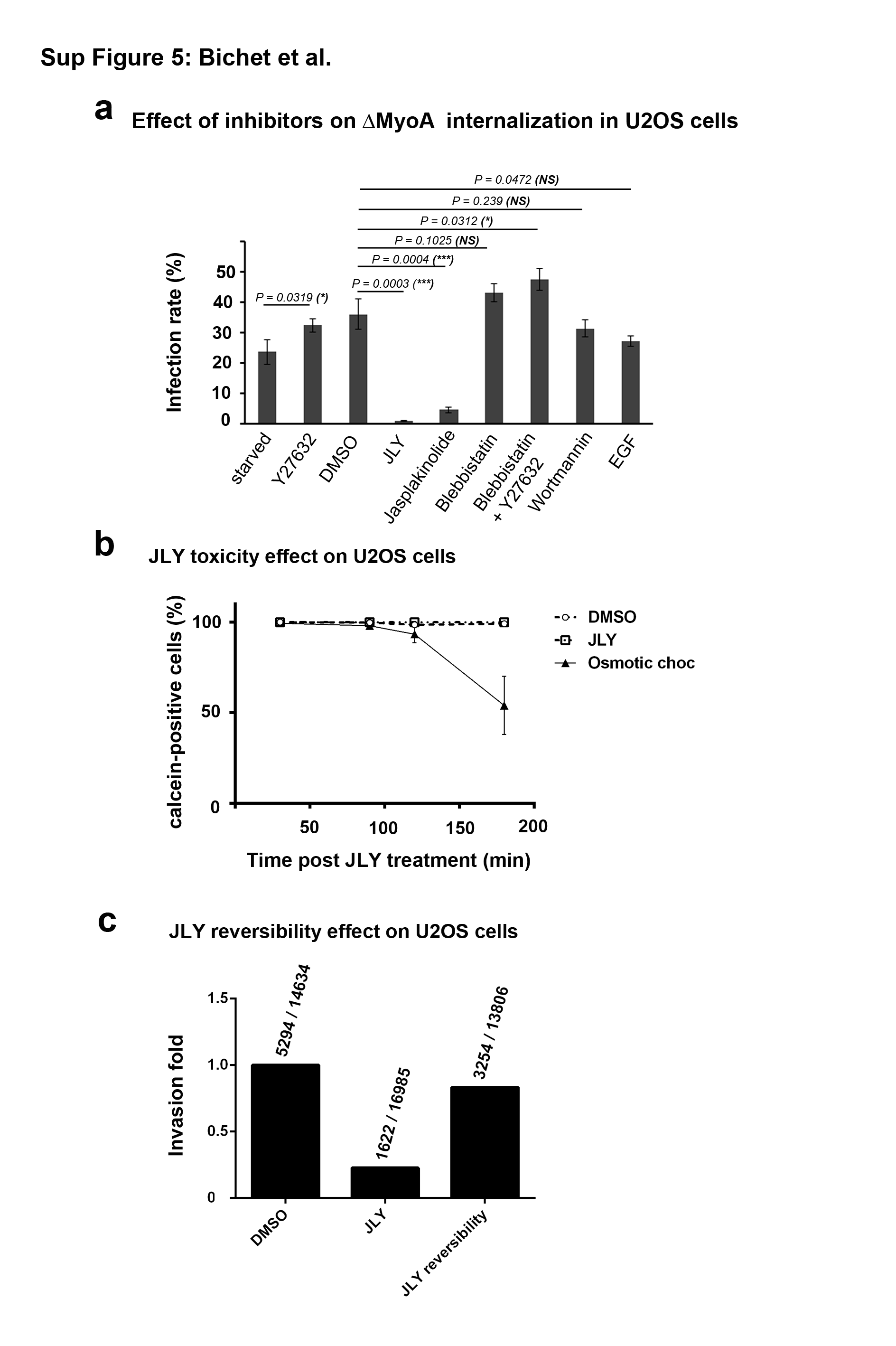

Supplement: Additional file 6: Figure S1. — (a) Histogram showing one representative invasion assay of U2OS cells by ΔMyoA tachyzoites under different drug treatment. Student t test has been applied using GraphPad Prism software. (b) Graph showing the amount of calcein-positive U2OS cells following JLY treatment and throughout a 2-h period to assess the cytotoxicity of the JLY treatment. Total cells are quantified by use of the cell permeant DNA stain Hoechst 33258. (c) Histogram showing the internalization index after normalization to control solvent conditions following (1) JLY treatment and (2) a 2-h recovery time post- JLY treatment to assess the reversibility of the drug effects. Numbers of cells infected versus total cells are indicated on top of each column. (TIF 282 kb) [file 12915_2016_316_MOESM6_ESM.tif]
